# Association between sarcopenic obesity and mortality in patients on peritoneal dialysis: a prospective cohort study

**DOI:** 10.3389/fmed.2024.1342344

**Published:** 2024-02-21

**Authors:** Yiwei Shen, Xinyu Su, Zanzhe Yu, Hao Yan, Dahua Ma, Yimei Xu, Jiangzi Yuan, Zhaohui Ni, Leyi Gu, Wei Fang

**Affiliations:** ^1^Department of Nephrology, Renji Hospital, School of Medicine, Shanghai Jiao Tong University, Shanghai, China; ^2^Shanghai Center for Peritoneal Dialysis Research, Shanghai, China; ^3^Jiangsu Hengrui Pharmaceuticals Co., Ltd., Shanghai, China

**Keywords:** sarcopenic obesity, sarcopenia, prevalence, survival, peritoneal dialysis

## Abstract

**Background:**

Whether sarcopenic obesity had unfavorable effect on survival of peritoneal dialysis (PD) patients is unknown. We aimed to investigate the association between sarcopenic obesity and survival in PD patients.

**Methods:**

This was a prospective observational study. Eligible PD patients from November 2016 to December 2017 were enrolled and followed until August 31, 2023. Sarcopenia was defined following the recommendations of the Asian Working Group for Sarcopenia (AWGS) as low appendicular skeletal muscle mass index (ASMI) and handgrip strength (HGS). Obesity was defined using the percentage of body fat (PBF). Survival analysis was conducted using the Kaplan–Meier and log-rank test. The Cox regression and the cumulative incidence competing risk (CICR) analyzes were used to investigate the association between sarcopenic obesity and all-cause mortality.

**Results:**

A total of 223 patients were enrolled with 133 (59.6%) males, a median age of 57.5 (44.6, 65.7) years, a median dialysis vintage of 20.3 (6.4, 57.7) months and 48 (21.5%) who had comorbid diabetes mellitus. Among them, 46 (20.6%) patients were sarcopenic, and 25 (11.2%) patients were diagnosed with sarcopenic obesity. After followed up for 51.6 (25.6, 73.9) months, the Kaplan–Meier curve showed the sarcopenic obesity (log-rank = 13.527, *p* < 0.001) group had significant lower survival rate compared to the nonsarcopenic non-obesity group. For multivariate analysis, the CICR method showed patients with sarcopenic obesity had significantly higher mortality rate (HR: 2.190, 95% CI: 1.011–4.743, *p* = 0.047) compared to those with nonsarcopenic non-obesity.

**Conclusion:**

Sarcopenia is not uncommon in PD patients, with a considerable proportion having sarcopenic obesity. There is a significant association between sarcopenic obesity and an increased risk of mortality in PD patients.

## Introduction

Peritoneal dialysis (PD), one of the major kidney replacement therapies, has shown survival advantage among patients with end-stage kidney disease (ESKD) (1, 2). The incidence of PD initiation has increased rapidly worldwide in the last 10 years (3, 4). Factors such as the use of bio-incompatible dialysis solutions, accumulation of uremic toxins, and chronic inflammation may contribute to protein-energy waste, which refers to a state of insufficient nutrient intake, depleted energy reserves, disruptions in body composition, and heightened muscle protein breakdown in patients with ESKD (5). Consequently, it can result in the loss of lean body mass in this patient population (6). Sarcopenia, characterized by muscle atrophy and impaired muscle function, is strongly associated with adverse clinical outcomes in dialysis patients, including worse quality of life, higher hospitalization rate and increased mortality (7, 8). Similar to the trend in the general population, obesity is increasingly common in patients receiving PD (9). It has been shown that the prevalence of obesity has increased substantially after PD initiation (10). Daily access to glucose-based dialysis solutions, insulin resistance, lipid metabolism disorders and reduced physical activity are risk factors that contribute to obesity in long-term PD treatment. The coexistence of sarcopenia and obesity, namely sarcopenic obesity, has garnered increasing attention due to its association with poorer survival in both general and elderly populations (11, 12). As PD patients are susceptible to develop both sarcopenia and obesity, the clinical outcomes of this cross-linked pathology are of great interest.

Only a few studies have investigated the clinical impact of sarcopenic obesity on dialysis patients’ outcomes, and the findings have been inconsistent. In a study by Malhotra et al. (13), sarcopenic obesity showed no statistically significant association with mortality in 122 patients undergoing hemodialysis (HD). Conversely, in a study by Sabatino et al., it was found that among 212 HD patients, the group with sarcopenic obesity exhibited worse survival in comparison to their counterparts with nonsarcopenic non-obesity (14). Given that PD patients are particularly susceptible to sarcopenic obesity, it is obviously of clinical importance to understand the association between sarcopenic obesity and patient outcomes. Therefore, this study was designed to investigate the association between sarcopenic obesity and survival in patients undergoing PD.

## Methods

### Study design

This was a prospective observational study undertaken in Renji Hospital, School of Medicine, Shanghai Jiao Tong University. The study was approved by the Ethics Committee of Renji Hospital (protocol code: [2016]101 K). Written informed consent was obtained from all recruited patients.

### Patient enrollment

Stable patients receiving continuous ambulatory peritoneal dialysis (CAPD) between November 1, 2016 and December 31, 2017 were screened for eligibility. Patients were eligible if they were aged 18 to 80 years and treated on PD for at least three months. Exclusion criteria included: (1) amputations; (2) implantation of cardiac pacemakers and internal defibrillators; (3) concomitant with severe diseases such as active malignancy, severe cardiac diseases, etc.; (4) ongoing severe infection; (5) planned or ongoing pregnancy or lactation; (6) combined HD; (7) refusal to give a written consent. All enrolled patients received treatment with lactate-buffered glucose-based dialysis solutions (Dianeal^®^, Baxter) utilizing a twin-bag system. No additional solutions were administered alongside the glucose-based dialysis solutions.

### Data collection

Demographic data were collected at enrollment including age, gender, height, weight, dialysis vintage, blood pressure, etiology of ESKD and comorbidities. Age-adjusted Charlson comorbidity index (aCCI) scores were calculated (15). Body mass index (BMI) was calculated as weight divided by height squared. Cardiovascular disease (CVD) was defined as the presence of myocardial infarction, coronary artery bypass surgery, percutaneous coronary intervention, congestive heart failure, angina pectoris, cerebrovascular disease and peripheral vascular disease. Fasting venous blood was collected from each patient at enrollment for the measurement of laboratory data including hemoglobin, serum albumin, fasting blood glucose (FBG), glycated hemoglobin (HbA1c), total cholesterol, total triglyceride, low-density lipoprotein-cholesterol (LDL-c), high-density lipoprotein-cholesterol (HDL-c) and high sensitivity C-reactive protein (hsCRP). Standard peritoneal equilibration test was performed to calculate the 4-h dialysate-to-plasma concentration ratio for creatinine (D/Pcr) (16). Weekly total urea clearance (Kt/V_urea_) and creatinine clearance (Ccr) were estimated using standard methods (17). Residual renal function (RRF) was evaluated as the average clearance of 24-h urinary urea and creatinine (18). Normalized protein catabolic rate (nPCR) was calculated and normalized to standard body weight (19). Daily glucose exposure to dialysate was assessed according to dialysis prescriptions.

### Measurement of body composition and muscle strength

At enrollment, body composition including extracellular water (ECW), intracellular water (ICW) and fat tissue mass (FTM) were measured using bioimpedance spectroscopy (BCM, Fresenius Medical Care, Germany) for each patient. The measurements were performed with patients in a supine position with 2 L dialysate dwell. The appendicular skeletal muscle mass index (ASMI) was calculated according to the regression models developed by using bioimpedance spectroscopy (20), as the sum of skeletal muscle mass of arms and legs divided by height squared. These regression models have been shown to strongly correlate with magnetic resonance imaging (MRI) measures of limb muscle mass. The equations for ASMI (arms) and ASMI (legs) were as following: ASMI (arms) = 1.69 + 0.301 × ICW (L) + 0.603 (male) + 0.0234 × weight (kg) – 0.123 × age (year), ASMI (legs) = 4.53 + 0.5005 × ICW (L) + 1.535 (male) + 0.0839 × weight (kg) – 0.0483 × age (year). Percentage of body fat (PBF) was calculated as the ratio of FTM and weight. Muscle strength, defined by handgrip strength (HGS), was measured using a digital dynamometer (CAMRY EH101, Guangdong, China). Patients were directed to perform three measurements using their dominant hand with maximum force, and the largest HGS was recorded.

Patients with low ASMI and low HGS were classified as sarcopenia. Low ASMI and low HGS were defined according to the recommendations of Asian Working Group for Sarcopenia (AWGS), as ASMI <7.0 kg/m^2^ for men and < 5.7 kg/m^2^ for women, and HGS < 26 kg for men and < 18 kg for women (21). Obesity was defined as PBF ≥ 25% for men and ≥ 35% for women (22). Therefore, our study population was divided into four groups based on muscle and fat status: non-sarcopenia and non-obesity (reference), nonsarcopenic obesity, nonobese sarcopenia, and sarcopenic obesity.

### Follow-up

All enrolled participants were prospectively followed up from enrollment until death, transfer to permanent HD, kidney transplantation, loss to follow-up, transfer to other dialysis centers, or to the end of the study (August 31, 2023). The outcome measure in our study was patient survival.

### Statistical analysis

Data normality was measured using Kolmogorov–Smirnov test. Continuous variables with normal distribution were presented as mean ± standard deviation and compared using One-Way ANOVA. Continuous variables with unnormal distribution were presented as median and interquartile range and compared using Kruskal-Wallis test. The Bonferroni or Tukey comparison post-hoc test was used in between-group comparisons. Categorical variables were presented as frequencies and percentages and compared using Chi-square test. Multivariate logistic regression analysis was run to explore the association between sarcopenia, sarcopenic obesity, and clinical and demographic variables. The Kaplan–Meier and log-rank test, and the Cox regression analysis were used to perform survival analysis. In addition, we used the cumulative incidence competing risk (CICR) analysis to mitigate the effect of competing events on survival. Analyzes were performed using IBM SPSS 22.0 for Windows software and R Statistical Software (v4.2.3). All statistical analysis used a significance level of 0.05 (2-sided).

## Results

### Patient characteristics

As shown in Table 1, a total of 223 patients were enrolled, of whom 133 (59.6%) were males, with a median age of 57.5 (44.6, 65.7) years and a median dialysis vintage of 20.3 (6.4, 57.7) months. The prevalence of sarcopenia in the study cohort was 20.6%. Of them, 25 (11.2%) patients met the criteria for sarcopenic obesity. Sarcopenic patients were older, with higher aCCI scores and FBG, lower HGS and ASMI than nonsarcopenic patients (all *p* < 0.05). When compared to the reference group, the sarcopenic obesity group had higher BMI, PBF, ECW/ICW, hsCRP, HbA1c, and lower blood pressure, HDL-c and Ccr (all *p* < 0.05). With comparison to the nonobese sarcopenia group, the sarcopenic obesity group had higher BMI, PBF, ECW/ICW, hsCRP, and lower HDL-c, Kt/V_urea_, Ccr (all *p* < 0.05). No significant difference was observed in the dialysis vintage, proportion of diabetes mellitus, serum albumin, glucose exposure and RRF (all *p* > 0.05) across the four subgroups.

**Table 1:** Patient characteristics according to the groups based on muscle and fat status.

	Total (*n* = 223)	Reference (*n* = 129)	Nonsarcopenic obesity (*n* = 48)	Nonobese sarcopenia (*n* = 21)	Sarcopenic obesity (*n* = 25)	*p* value
Age (years)	57.5 (44.6, 65.7)	52.5 (38.4, 62.4)	60.4 (48.8, 66.9)^#&^	65.7 (57.9, 68.8)^#^	68.3 (61.5, 71.6)^#*^	< 0.001
Dialysis vintage (months)	20.3 (6.4, 57.7)	18.6 (6.0, 53.6)	23.5 (7.8, 53.8)	41.9 (7.5, 97.3)	42.4 (9.6, 74.2)	0.211
Gender (male) [n (%)]	133 (59.6)	83 (64.3)	31 (64.6)^&^	5 (23.8)^#^	14 (56.0)	0.005
BMI (kg/m^2^)	23.4 ± 3.1	22.6 ± 2.7	26.2 ± 3.1^#&^	21.1 ± 2.3	24.2 ± 2.4^#&*^	< 0.001
*Etiology of ESKD [n (%)]*						0.155
Chronic glomerulonephritis	87 (39.0)	57 (44.2)	18 (37.5)	8 (38.1)	4 (16.0)	
Diabetic nephropathy	32 (14.3)	12 (9.3)	9 (18.8)	3 (14.3)	8 (32.0)	
Hypertension	6 (2.7)	5 (3.9)	1 (2.1)	0	0	
Polycystic kidney disease	7 (3.1)	2 (1.6)	2 (4.2)	1 (4.8)	2 (8.0)	
Others	16 (7.2)	10 (7.8)	4 (8.3)	0	2 (8.0)	
Unknown	75 (33.6)	43 (33.3)	14 (29.2)	9 (42.9)	9 (36.0)	
Comorbidities [*n* (%)]						
Hypertension	173 (77.6)	102 (20.9)	38 (79.2)	16 (76.2)	17 (68.0)	0.665
Diabetes mellitus	48 (21.5)	22 (17.1)	12 (25.0)	4 (19.0)	10 (40.0)	0.072
CVD	36 (16.1)	18 (14.0)	9 (18.8)	5 (23.8)	4 (16.0)	0.657
Systolic pressure (mmHg)	143 (132, 155)	145 (136, 160)	142 (132, 154)	141 (136, 152)	134 (127, 145)^#^	0.015
Diastolic pressure (mmHg)	91 (84, 99)	95 (87, 102)	89 (80, 96)^#^	88 (84, 90)^#^	86 (78, 91)^#^	< 0.001
aCCI	4 (3, 5)	3 (2, 4)	4 (3, 5)^#^	4 (3.5, 5)^#^	5 (4, 6)^#*^	< 0.001
Hemoglobin (g/L)	107.2 ± 16.7	104.9 ± 15.4	108.5 ± 19.6	113.8 ± 17.9	110.5 ± 14.5	0.071
Serum albumin (g/L)	38.9 ± 3.8	38.8 ± 3.9	39.7 ± 3.6	37.9 ± 4.4	38.2 ± 3.4	0.255
FBG (mmol/L)	4.7 (4.3, 5.6)	4.6 (4.3, 5.2)	4.8 (4.3, 6.2)	5.0 (4.4, 7.0)^#^	5.1 (4.8, 7.3)^#^	0.016
Glycated hemoglobin (%)	5.3 (5.0, 5.8)	5.2 (4.9, 5.8)	5.5 (4.9, 6.0)	5.3 (5.2, 5.7)	5.6 (5.3, 6.7)^#^	0.012
Total cholesterol (mmol/L)	4.89 ± 1.16	4.84 ± 1.09	4.71 ± 1.12^&^	5.59 ± 1.38^#^	4.94 ± 1.21	0.027
Total triglycerides (mmol/L)	1.67 (1.12, 2.29)	1.61 (1.03, 2.14)	1.76 (1.27, 3.00)	1.76 (1.21, 2.23)	1.68 (1.22, 3.14)	0.361
HDL-c (mmol/L)	1.08 (0.85, 1.33)	1.11 (0.86, 1.33)	0.96 (0.79, 1.10)^#&^	1.39 (1.14, 1.57)^#^	0.97 (0.76, 1.16)^#&^	< 0.001
LDL-c (mmol/L)	2.96 ± 0.94	2.92 ± 0.95	2.84 ± 0.86	3.42 ± 1.03	3.02 ± 0.93	0.107
HsCRP (mg/L)	1.58 (0.62, 5.22)	1.22 (0.55, 4.65)	2.31 (0.63, 5.64)	1.58 (0.55, 2.66)	4.07 (1.20, 13.5)^#&^	0.012
Glucose exposure (g/d)	130 (110, 180)	130 (97.5, 160)	140 (110, 180)	110 (90, 155)	160 (110, 190)	0.075
4-h D/Pcr	0.60 (0.52, 0.69)	0.61 (0.54, 0.70)	0.61 (0.50, 0.68)	0.59 (0.43, 0.73)	0.58 (0.53, 0.65)	0.385
Kt/V_urea_	1.85 (1.57, 2.13)	1.86 (1.58, 2.17)	1.70 (1.46, 1.93)^#&^	2.06 (1.88, 2.50)^#^	1.85 (1.59, 2.04)^&^	0.004
Ccr	57.28 (48.53, 70.25)	58.48 (49.60, 73.28)	51.85 (45.91, 64.14)^#&^	61.86 (51.57, 79.70)	52.57 (47.94, 62.11)^#&^	0.012
RRF (ml/min)	1.30 (0, 3.57)	1.75 (0, 3.79)	0.71 (0, 3.49)	1.45 (0.38, 4.25)	0.49 (0, 1.63)	0.158
nPCR (g/kg/d)	0.94 ± 0.20	0.96 ± 0.19	0.86 ± 0.20^#&^	1.05 ± 0.16	0.91 ± 0.21	0.001
HGS (kg)	24.9 (18.5, 31.9)	27.4 (21.4, 34.4)	26.5 (19.7, 34.5)^&^	16.6 (12.4, 17.7)^#^	16.1 (14.2, 19.9)^#*^	< 0.001
ASMI (kg/m^2^)	7.1 (5.7, 8.1)	7.5 (6.5, 8.4)	7.2 (6.1, 8.0)^&^	5.2 (4.9, 5.6)^#^	5.5 (4.7, 6.1)^#*^	< 0.001
PBF (%)	27.4 (20.0, 33.0)	20.9 (16.1, 27.7)	34.2 (30.0, 36.8)^#&^	26.7 (20.7, 30.9)	36.2 (33.0, 40.4)^#&^	< 0.001
ECW/ICW	0.92 ± 0.12	0.87 ± 0.10	0.96 ± 0.10^#^	0.94 ± 0.12	1.04 ± 0.15^#&*^	< 0.001

Variables were presented as mean ± standard deviation, median and interquartile range, frequencies and percentages and compared using One-Way ANOVA, Kruskal-Wallis test and Chi-square test, followed by Bonferroni or Tukey comparison post-hoc test.

BMI, body mass index; ESKD, end-stage kidney disease; CVD, Cardiovascular disease; aCCI, age-adjusted Charlson comorbidity index; FBG, Fasting blood glucose; HDL-c, high-density lipoprotein-cholesterol; LDL-c, low-density lipoprotein-cholesterol; HsCRP, high sensitivity C-reactive protein; D/Pcr, dialysate-to-plasma concentration ratio for creatinine; Ccr, total creatinine clearance; RRF, residual renal function; nPCR, normalized protein catabolic rate; HGS, handgrip strength; ASMI, appendicular skeletal muscle mass index; PBF, percentage of body fat; ECW, extracellular water; ICW, intracellular water.

^#^ *p* < 0.05 vs. reference (nonsarcopenic non-obesity); ^&^ *p* < 0.05 vs. nonobese sarcopenia; ^*^ *p* < 0.05 vs. nonsarcopenic obesity.

Age (OR = 1.145, 95% CI: 1.083–1.210, *p* < 0.001), ECW/ICW (OR = 1.091, 95% CI: 1.035–1.150, *p* = 0.001) and BMI (OR = 0.701, 95% CI: 0.585–0.839, *p* < 0.001) were significantly associated with sarcopenia after adjustment for gender, dialysis vintage, glucose exposure, serum albumin, total cholesterol, hsCRP, Kt/V_urea_, RRF and diabetes mellitus. Age (OR = 1.096, 95% CI: 1.033–1.162, *p* = 0.002) and ECW/ICW (OR = 1.101, 95% CI: 1.040–1.166, *p* = 0.001) were significantly associated with sarcopenic obesity when adjusting for gender, dialysis vintage, glucose exposure, serum albumin, total cholesterol, hsCRP, Kt/V_urea_, RRF and diabetes mellitus (Table 2).

**Table 2:** Multivariate logistic regression of the association between sarcopenia, sarcopenic obesity and clinical and demographic variables.

	Sarcopenia			Sarcopenic obesity		
	OR	95% CI	*p* value	OR	95% CI	*p* value
Age	1.145	1.083–1.210	<0.001	1.096	1.033–1.162	0.002
Gender (male)	0.503	0.148–1.706	0.270	1.287	0.312–5.305	0.727
Dialysis vintage	1.010	0.996–1.024	0.161	0.995	0.978–1.011	0.507
Glucose exposure	0.991	0.978–1.006	0.236	0.994	0.979–1.010	0.480
Serum albumin	1.109	0.971–1.266	0.126	1.141	0.981–1.327	0.086
Total cholesterol	1.339	0.899–1.996	0.151	0.981	0.639–1.507	0.931
HsCRP	1.015	0.969–1.064	0.528	1.045	0.995–1.098	0.076
Kt/V_urea_	1.918	0.392–9.372	0.421	2.300	0.396–13.377	0.354
RRF	0.937	0.644–1.364	0.735	0.800	0.530–1.207	0.288
Diabetes mellitus	1.621	0.576–4.560	0.360	1.018	0.317–3.269	0.976
BMI	0.701	0.585–0.839	<0.001	0.945	0.796–1.121	0.516
ECW/ICW (increase 0.01)	1.091	1.035–1.150	0.001	1.101	1.040–1.166	0.001

OR, odds ratio; CI, confidence interval; BMI, body mass index; HsCRP, high sensitivity C-reactive protein; Kt/V_urea_, weekly total urea clearance; RRF, residual renal function; ECW, extracellular water; ICW, intracellular water.

### Patient outcomes

As shown in Table 3, after followed up for 51.6 (25.6, 73.9) months, 60 (26.9%) patients died, 59 (26.5%) patients transferred to permanent HD, 18 (8.1%) patients underwent kidney transplantation, 8 (3.6%) patients transferred to other centers, 3 (1.3%) patients were lost to follow-up, and 75 (33.6%) patients were still on PD. The leading cause of death was cardiovascular events for 38 (63.3%) patients. No significant difference was found in the distribution of outcomes among four subgroups.

**Table 3:** Outcomes of patients.

	Total (*n* = 223)	Reference (*n* = 129)	Nonsarcopenic obesity (*n* = 48)	Nonobese sarcopenia (*n* = 21)	Sarcopenic obesity (*n* = 25)	*p* value
Follow-up (months)	51.6 (25.6, 73.9)	56.6 (30.0, 74.6)	50.5 (22.3, 65.8)	40.4 (10.0, 74.9)	50.1 (29.8, 77.2)	0.404
*Outcomes [n (%)]*						0.134
Death	60 (26.9)	22 (17.1)	18 (37.5)	7 (33.2)	13 (52.0)	
Transfer to hemodialysis	59 (26.5)	37 (28.7)	11 (22.9)	6 (28.6)	5 (20.0)	
Kidney transplantation	18 (8.1)	14 (10.8)	3 (6.3)	1 (4.8)	0	
Transfer to other centers	8 (3.6)	5 (3.9)	2 (4.2)	1 (4.8)	0	
Lost to follow-up	3 (1.3)	2 (1.5)	1 (2.1)	0	0	
Still on PD	75 (33.6)	49 (38.0)	13 (27.1)	6 (28.6)	7 (28.0)	
Death [*n* (%)]	*N* = 60	*N* = 22	*N* = 18	*N* = 7	*N* = 13	
Cardiovascular events	38 (63.3)	14 (63.6)	11 (61.1)	5 (71.4)	8 (61.5)	
Cardiac disease	15 (25.0)	3 (13.6)	5 (27.8)	2 (28.6)	5 (38.5)	
Cerebrovascular disease	13 (21.7)	7 (31.8)	3 (16.7)	2 (28.6)	1 (7.7)	
Peripheral vascular disease	3 (5.0)	1 (4.5)	1 (5.6)	0	1 (7.7)	
Sudden death	7 (11.7)	3 (13.6)	2 (11.1)	1 (14.3)	1 (7.7)	
Infection	11 (18.3)	5 (22.7)	2 (11.1)	0	4 (30.8)	
Peritonitis	4 (6.7)	2 (9.1)	2 (11.1)	0	0	
Pneumonia	5 (8.3)	2 (9.1)	0	0	3 (23.1)	
Sepsis	2 (3.3)	1 (4.5)	0	0	1 (7.7)	
Malignancy	3 (5.0)	1 (4.5)	2 (11.1)	0	0	
Gastrointestinal hemorrhage	2 (3.3)	1 (4.5)	1 (5.6)	0	0	
Unknown	6 (10.0)	1 (4.5)	2 (11.1)	2 (28.6)	1 (7.7)	

Variables were presented as median and interquartile range, and frequencies and percentages and compared using Kruskal-Wallis test and Chi-square test. PD, peritoneal dialysis.

### Patient survival and association between all-cause mortality and groups based on muscle and fat status

**Figure 1:**
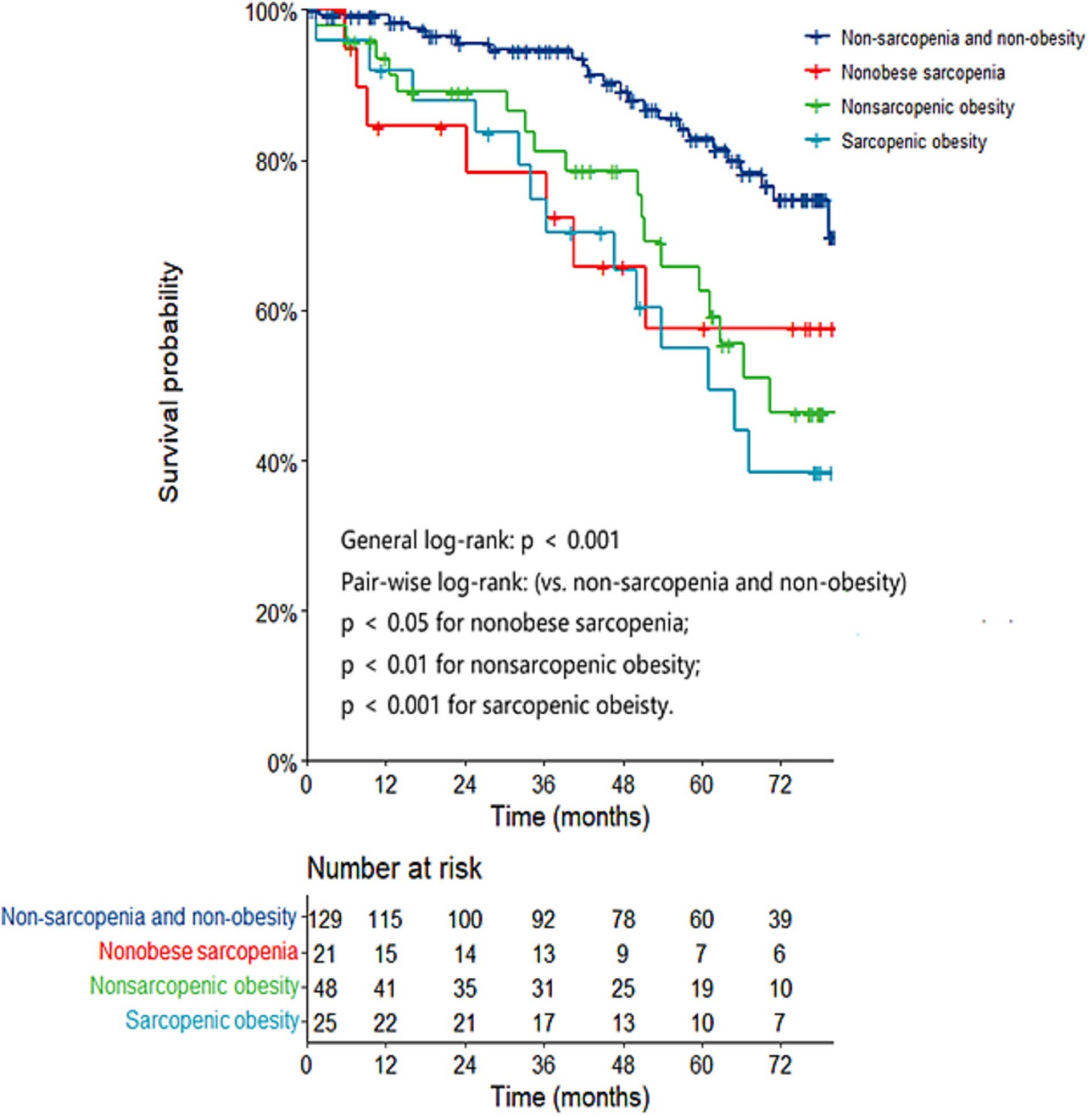
Kaplan–Meier curves based on muscle and fat status for patient survival.

As shown in Figure 1, the sarcopenic obesity (log-rank = 13.527, *p* < 0.001) and the nonobese sarcopenia groups (log-rank = 4.830, *p* = 0.028) had significantly inferior survival in comparison to the reference group. Among the patients with sarcopenia, no significant difference was found in survival between the sarcopenic obesity group and the nonobese sarcopenia group (log-rank = 0.310, *p* = 0.578).

In the multivariate Cox regression analysis, the nonobese sarcopenia (*p* = 0.191) and the sarcopenic obesity groups (*p* = 0.139) were no longer associated with all-cause mortality compared to the reference group when adjusting for gender, dialysis vintage, serum albumin, total cholesterol, hsCRP and aCCI. However, when the CICR model was used to mitigate the effect of censored events, the sarcopenic obesity group was independently associated with increased all-cause mortality compared to the reference group (HR: 2.190, 95% CI: 1.011–4.743, *p* = 0.047) (Table 4).

**Table 4:** Multivariate Cox regression and CICR analyzes for all-cause mortality according to groups based on muscle and fat status.

	Univariate		Multivariate			
	cs-HR (95% CI)	*p* value	cs-HR (95% CI)	*p* value	sd-HR (95% CI)	*p* value
Gender (male)	1.119 (0.661–1.894)	0.677	1.030 (0.582–1.823)	0.920	1.175 (0.661–2.086)	0.58
Dialysis vintage	0.999 (0.991–1.006)	0.730	0.996 (0.987–1.006)	0.433	0.996 (0.989–1.004)	0.35
aCCI	1.558 (1.322–1.836)	< 0.001	1.469 (1.208–1.786)	< 0.001	1.491 (1.235–1.800)	<0.001
Serum albumin	0.929 (0.876–0.984)	0.013	0.924 (0.867–0.986)	0.017	0.925 (0.867–0.986)	0.016
HsCRP	1.016 (0.990–1.043)	0.224	1.000 (0.971–1.029)	0.987	0.993 (0.961–1.026)	0.67
Total cholesterol	1.186 (0.933–1.509)	0.164	1.124 (0.870–1.451)	0.371	1.075 (0.818–1.414)	0.60
Anuria	1.542 (0.904–2.627)	0.112	1.871 (0.916–3.821)	0.085	1.630 (0.784–3.387)	0.19
Groups based on muscle and fat status						
Nonsarcopenic non-obesity	Reference		Reference		Reference	
Nonobese sarcopenia	2.521 (1.076–5.906)	0.033	1.888 (0.728–4.898)	0.191	1.872 (0.652–5.374)	0.24
Nonsarcopenic obesity	2.567 (1.374–4.795)	0.003	2.134 (1.100–4.141)	0.025	2.269 (1.170–4.400)	0.015
Sarcopenic obesity	3.356 (1.690–6.665)	0.001	1.787 (0.828–3.859)	0.139	2.190 (1.011–4.743)	0.047

CICR, cumulative incidence competing risk; cs-HR, cause-specific hazard ratio; sd-HR, subdistribution hazard ratio; CI, confidence interval; aCCI, age-adjusted Charlson comorbidity index; HsCRP, high sensitivity C-reactive protein; RRF, residual renal function. Anuria was defined as urine output <100 mL/d.

## Discussion

The present study showed that sarcopenia was not uncommon in PD patients, with a significant proportion of them having sarcopenic obesity. The group with sarcopenic obesity was independently associated with increased mortality in PD patients compared to the nonsarcopenic nonobese group. However, among patients with sarcopenia, there was no significant difference in survival between patients with nonobese sarcopenia and sarcopenic obesity.

The prevalence of sarcopenia and sarcopenic obesity in ESKD patients varies significantly depending on the diagnostic criteria, assessment tools and cut-off values used. Previous studies showed that the prevalence of sarcopenia and sarcopenic obesity in patients on PD ranging from 4 to 38.2%, and from 3 to 8.6%, respectively (23–27). Similarly, a prevalence of 20.6% for sarcopenia and 11.2% for sarcopenic obesity was reported in our study cohort. These findings suggested that sarcopenia and sarcopenic obesity were not uncommon in PD patients.

In the present study, we observed a significant association between sarcopenic obesity and advanced age, as well as higher ECW/ICW ratios. Age is widely recognized as an important risk factor for sarcopenic obesity in the general population (28). Elderly PD patients are particularly vulnerable to sarcopenia due to factors such as uremic toxins, dialysis treatment and dietary restrictions. They also tend to gain fat more easily due to exposure to glucose-based dialysis solutions and reduced physical activities. Consistent with our findings, Sabatino et al. conducted a retrospective study involving 212 HD patients and found age was independently associated with sarcopenic obesity (OR = 1.17, 95% CI: 1.09–1.25, *p* < 0.001). Therefore, it is crucial to pay close attention to the evaluation of sarcopenic obesity in elderly individuals undergoing dialysis. It has been indicated that there might be a strong association between volume status and nutritional status (29). Recent studies have suggested that the ECW/ICW ratio may be a more sensitive indicator of changes in muscle properties than muscle mass alone (30). While the exact mechanism underlying the interaction between volume overload and sarcopenic obesity in ESKD patients is not yet fully understood, it is speculated that chronic inflammation resulting from excessive fluid (31) might contribute to both the rise in ECW/ICW ratios and the development of sarcopenic obesity. In general, these findings suggested that sarcopenic obesity was more likely to occur in patients who were older and experiencing volume overload.

Regarding patient survival, we found that the sarcopenic obesity group had poorer survival than that with nonsarcopenic non-obesity. Several retrospective studies have consistently reported a strong association between sarcopenic obesity and an elevated risk of mortality among PD patients. Do and Kang (32) retrospectively evaluated the association between sarcopenia or its components, obesity and patient survival by analyzing the body composition of 199 incident PD patients. After 18 months of follow-up, they reported that the survival rate of patients with nonsarcopenic non-obesity, nonobese sarcopenia, and sarcopenic obesity were 97.5, 83.5, and 70.1%, respectively. Furthermore, patients with sarcopenic obesity had significantly lower survival rate compared to those with nonsarcopenic non-obesity. Another study conducted in Italy, which utilized abdominal computed tomography to diagnose sarcopenia and obesity in 212 HD patients, reported that the group with sarcopenic obesity exhibited inferior survival in comparison to the group with nonsarcopenic non-obesity (14). The present prospective study showed that sarcopenic obesity served as an independent predictor for higher mortality rate in PD patients, which was in accordance with previous studies.

Obesity is known to be an important risk factor for cardiovascular disease and increased mortality in the general population (33). However, there is evidence of a reverse epidemiology phenomenon referred to as the “obesity paradox” in ESKD patients, wherein patients with obesity may experience improved clinical outcomes (34). In a study conducted in a 2-year cohort of 54,535 HD patients in the United States, obesity was described to be associated with reduced cardiovascular mortality significantly (35). Moreover, another study enrolled 261 HD patients reported better quality of life and lower risk of all-cause mortality and cardiovascular risk in patients with sarcopenic obesity compared to those with nonobese sarcopenia (36). However, our study did not identify a survival advantage in the sarcopenic obesity group compared to the nonobese sarcopenia group, which aligned with the findings of studies conducted by Do and Kang (32) as well as Sabatino (14). Possible explanations for the varying results could be attributed to the different dialysis modalities, longer dialysis vintage, extended follow-up period and better nutritional status in our cohort. Body fat is thought to have positive effect on nutrition in the short term by serving as source of energy (34, 37). But excess body fat is known to be strongly associated with inflammation (38), which may have an adverse effect on survival in the long term, as supported by our finding that the sarcopenic obesity patients had higher hsCRP levels. Taken together, the reverse epidemiology phenomenon of obesity may be not present in patients on PD with a long dialysis vintage and favorable nutritional status.

Our study has several limitations. Firstly, due to the single-center design, the results might not be extrapolated to patients in other populations. Secondly, we did not conduct longitudinal monitoring of patients’ muscle and adipose tissue, thus limiting our understanding of the association between patient outcomes and changes in muscle and fat status. Thirdly, patients older than 80 were excluded in the present study, who may exhibit a higher prevalence of sarcopenic obesity. Fourthly, we lacked information regarding patient daily activity and personal habits such as smoking and alcohol consumption, which are known to be associated closely with patients’ outcomes. Fifthly, we did not document new onset hypertension, diabetes mellitus, and dyslipidemia during the 6-year follow-up, despite their potential impact on patient prognosis. Sixthly, the muscle and fat values obtained from bioimpedance analysis were not compared with those from other body composition assessment tools, such as muscle and fat ultrasound. The application of ultrasound technique has been reported to offer reliable and valid measurements of muscle and fat mass, and their parameters have shown significant correlation with the results of bioimpedance analysis (39, 40). Clearly, multicenter studies with better design and larger sample size are needed to confirm our results.

## Conclusion

In summary, our results showed that sarcopenia, and in particular sarcopenic obesity, was not uncommon in PD patients. Sarcopenic obesity was significantly associated with increased mortality risk in PD patients. Therefore, we suggest attention should be directed towards this interconnected pathology, rather than solely focusing on alterations in muscle or adipose tissue.

## Data availability statement

The original contributions presented in the study are included in the article/supplementary material, further inquiries can be directed to the corresponding author.

## Ethics statement

The studies involving humans were approved by Ethics Committee of Renji Hospital, School of Medicine, Shanghai Jiao Tong University, Shanghai, China. The studies were conducted in accordance with the local legislation and institutional requirements. The participants provided their written informed consent to participate in this study.

## Author contributions

YS: Conceptualization, Formal analysis, Investigation, Writing – original draft, Writing – review & editing, Visualization. XS: Investigation, Writing – original draft. ZY: Investigation, Writing – original draft. HY: Investigation, Writing – original draft. DM: Investigation, Writing – original draft. YX: Investigation, Writing – original draft. JY: Writing – review & editing. ZN: Writing – review & editing. WF: Funding acquisition, Investigation, Project administration, Resources, Supervision, Writing – review & editing, Conceptualization, Writing – original draft, Visualization, Validation. LG: Resources, Visualization, Validation, Writing – review & editing.

## Glossary

PD: peritoneal dialysis
ESKD: end-stage kidney disease
HD: hemodialysis
aCCI: age-adjusted Charlson comorbidity index
BMI: body mass index
CVD: cardiovascular disease
FBG: fasting blood glucose
HbA1c: glycated hemoglobin
LDL-c: low-density lipoprotein-cholesterol
HDL-c: high-density lipoprotein-cholesterol
hsCRP: high sensitivity C-reactive protein
D/Pcr: dialysate-to-plasma concentration ratio for creatinine
Kt/V_urea_: weekly total urea clearance
Ccr: creatinine clearance
RRF: residual renal function
nPCR: normalized protein catabolic rate
ECW: extracellular water
ICW: intracellular water
FTM: fat tissue mass
ASMI: appendicular skeletal muscle mass index
PBF: percentage of body fat
HGS: handgrip strength
AWGS: Asian Working Group for Sarcopenia
CICR: cumulative incidence competing risk
OR: odds ratio
CI: confidence interval
cs-HR: cause-specific hazard ratio
sd-HR: subdistribution hazard ratio
